# Association of Antibiotic Resistance Traits in Uropathogenic *Escherichia coli* (UPEC) Isolates

**DOI:** 10.1155/2022/4251486

**Published:** 2022-03-16

**Authors:** Md. Mostafizer Rahman, Mridha Md. Kamal Hossain, Rubaya Rubaya, Joyanta Halder, Md. Ekramul Karim, Anjuman Ara Bhuiya, Anwara Khatun, Jahangir Alam

**Affiliations:** ^1^Department of Microbiology, Gono Bishwabidyalay, Dhaka, Bangladesh; ^2^National Institute of Biotechnology, Dhaka, Bangladesh; ^3^Department of Microbiology, Jagannath University, Dhaka, Bangladesh; ^4^Department of Biology and Biochemistry, University of Houston, Texas, USA

## Abstract

**Background:**

Antimicrobial resistance (AMR) is a global health problem which is constantly evolving and varies spatially and temporally. Resistance to a particular antibiotic may serve as a selection and coselection marker for the same or different antibiotic classes. Therefore, this cross-sectional study was conducted to predict the association of phenotypic and genotypic resistance traits in uropathogenic *Escherichia* coli (UPEC).

**Method:**

A total of 42 UPEC from 83 urine samples were investigated for the prevalence and association of phenotypic and genotypic AMR traits. Antibiogram profiling was carried out by Kirby–Bauer's disc diffusion method and AMR genes (ARGs) were detected by PCR.

**Result:**

UPECs were isolated from 50.60% (42/83) of the samples examined. Of these, 80.95% of cases were derived from females, and 38.10% of cases were found in the age group of 21–30 years. The isolates were shown to have a high frequency of resistance to tetracycline (92.86%), followed by sulfonamide (71.43%), ampicillin (52.38%), trimethoprim-sulfamethoxazole (47.62%), and 28.57% each to streptomycin, chloramphenicol, and erythromycin. The most prevalent antimicrobial resistance genes (ARGs) in these isolates were *tet*(A) (78.57%), *tet*(B) (76.19%), *sul*1 (61.91%), *dfr*A1 (35.71%), *bla*_SHV_ (26.19%), *cml*A (19.05%), and CITM, *qnrA*, and *cat*A1 each at 11.91%. According to statistical analysis, ampicillin, sulfonamide, trimethoprim-sulfamethoxazole, and ciprofloxacin resistance were strongly correlated with the presence of *bla*_SHV_, *sul*1, *dfr*A1, and *qnr*A, respectively. Nonsignificant associations were observed between ciprofloxacin-tetracycline, sulfonamide-erythromycin pairs as well as between *tet*(A) and *tet*(B) genes. Besides, coselection was also assumed in the case of chloramphenicol resistance genes, namely, *cat*A1 and *cml*A.

**Conclusion:**

Both the phenotypic and genetic resistance traits were found in the UPEC isolates. Statistical association and coselection phenomena among AMR phenotypes and genotypes were also observed but required to be validated in a broad-scale study. However, these findings might have important implications for the development of an AMR prediction model to tackle future AMR outbreaks.

## 1. Introduction

Antimicrobial resistance (AMR) is one of the world's most urgent public health concerns. The increasing phenomena of AMR has been continuously affecting healthcare, veterinary, and agricultural settings globally which ultimately endangers the achievement of sustainable development goals [1]. Coselection and persistence of resistance to common and critically important antimicrobial drugs are occurring due to massive and inappropriate antibiotic use. This in turn results in the continuous evolution and spread of multidrug-resistant (MDR) pathogens [2]. Due to the emergence/reemergence of MDR pathotypes, the empirical treatment in clinical settings is becoming challenging.

Resistant bacteria often serve as reservoir of diverse antimicrobial resistance genes (ARGs) [3]. ARGs located on mobile genetic elements may get transferred to other bacteria of clinical significance, mostly through horizontal gene transfer mechanisms [4]. *Escherichia coli* is one of the most suitable candidate vehicles for such gene transfer because of its diverse presence in environmental niches as well as a common flora in the gastrointestinal tracts of both humans and animals [5]. They are sensitive to selection pressure exerted by antibiotic usage and carry genetic mobile elements to achieve such transmission [4]. For this reason, multidrug resistance in *E. coli* is increasingly observed in human healthcare settings as well as in veterinary medicine globally. AMR and ARGs of *E. coli* have been reported previously and known to harbor numerous drug-resistance phenotypes and genotypes such as the betalactam resistance (*bla*_TEM-1_, *bla*_SHV_, *bla*_CTX-M_, and *bla*_NDM-1_), tetracycline resistance (*tet*(A), *tet*(B), *tet*(C), and *tet*(H)), sulfonamide-trimethoprim resistance (*sul*1, *sul*2, and *dfr*), aminoglycosides resistance (*aac*(3)–II/IV and *aac*(6)-Ib), phenicol resistance (*cml*A and *cat*A1), and fluoroquinolones resistance (*qnrA*, *qnrB*, and *qnr*S) [6]. Among the *E. coli* pathotypes, uropathogenic *E. coli* (UPEC) is responsible for the majority (70–90%) of urinary tract infections (UTIs) [7]. UTIs can be community- or nosocomial-acquired where UPEC strains are reported to cause 70–95% of community-acquired and 50% of nosocomial-acquired cases [8]. AMR phenotypes may arise from different genetic determinants, each of which may present specific epidemiological features [9]. Therefore, surveillance of antimicrobial resistance from phenotypic and genotypic perspectives in bacterial populations can be useful to predict AMR outcomes. In addition, this type of study represents a potentially useful tool to extend the understanding of AMR epidemiology for future outbreak prediction and judicious choice of antibiotics.

UTIs are one of the most frequent human infections worldwide including Bangladesh occurring in people of all ages and sex, but more commonly in women and elderly patients. Despite numerous studies on the AMR of *E. coli* from diverse niches such as veterinary, environmental, and clinical settings [9–14], there is a scarcity of information on the association between AMR phenotypes and ARGs and their coselection phenomena in UPEC isolates. Therefore, the study was designed to investigate the prevalence and distribution of AMR phenotypes and ARGs in UPEC isolates. In addition, attempt was made to predict the association as well as coselection phenomena among the AMR phenotypes and ARGs.

## 2. Materials and Methods

### 2.1. Study Design

This cross-sectional study was conducted during August 2016–June 2017. Urine samples of UTI-suspected patients were collected and examined for uropathogenic *E. coli* (UPEC). Only confirmed UPEC associated UTI patients' relevant data were considered for this study.

### 2.2. Media and Chemicals

Blood agar, MacConkey agar, and Eosin-methylene blue (EMB) agar were used for selective isolation and identification of UPEC, whereas Mueller–Hinton agar (MHA) was used for antibiotic susceptibility test. All the media and standard antibiotic discs were purchased from Oxoid Ltd., UK. Molecular biology grade reagents for DNA extraction, PCR, and gel electrophoresis were purchased from Invitrogen, USA. Oligonucleotide probes for 16S rRNA gene amplification were synthesized by Macrogen Inc., South Korea, and collected by a local distributor.

### 2.3. Sampling and Microbiological Analysis

Clean catch midstream urine samples were collected from patients, irrespective of age and sex, reporting at the outpatient department of Gonoshasthaya Kendra, Dhaka, Bangladesh, according to the Clinical and Laboratory Standard Institute guidelines [15]. Patients were provided with properly labelled wide-mouthed sterile leak-proof screw cap containers and requested to collect urine aseptically. A total of 83 urine samples were collected randomly and used for the isolation and identification of UPEC. For this purpose, about 100 µL of each sample was inoculated onto sterilized 5% blood agar and MacConkey agar plates and incubated at 37°C for 24 h. Following overnight incubation, positive bacteriuria cases have been confirmed where 10^5^ cfu/ml colony counts were recorded [16]. Furthermore, *E. coli* isolates were randomly selected and isolated from each of the positive bacteriuria cases following repeated streaking on MacConkey agar and EMB agar based on cultural characteristics and preserved in 50% LB-glycerol broth at −80°C for further analysis.

### 2.4. Identification of *E. coli*

Selected UPEC isolates were phenotypically characterized based on their cultural, morphological, and biochemical characteristics following the taxonomic guidelines of the District Laboratory Practice in Tropical Countries [17]. Molecular identification of the isolates was confirmed by PCR. For PCR analysis, bacterial genomic DNA was extracted from an overnight-grown *E. coli* culture using the TIANamp Genomic DNA extraction kit (TIANGEN Biotech. Co. Ltd., China) according to the manufacturer's instructions. The purity of the extracted DNA was checked by NanoDrop™ 1000 Spectrophotometer (Thermo Fisher Scientific, USA) and stored at −20°C for further analysis. PCR amplification was performed using previously reported primers (16E1-F: 5′-GGGAGTAAAGTTAATCCTTTGCTC-3′ and 16E2-R: 5′-TTCCCGAAGGCACATTCT-3′) targeting 584 bp fragment of 16S rRNA gene [18]. A PCR reaction was performed in a total volume of 25 *μ*L, containing 1.5 mM MgCl_2_, 50 mM KCl, 10 mM Tris-HCl (pH 9.0), 0.1% Triton X-100, 200 *μ*M of each dNTP, 1 *μ*M of primers, 1 unit of Taq DNA polymerase, and 5 *μ*L (∼50 ng/*μ*L) of genomic DNA in the Gene Atlas thermocycler (ASTEC, Japan). The PCR conditions included an initial denaturation for 5 min at 95°C, followed by 35 cycles consisting of denaturation at 94°C for 1 min, annealing at 55°C for 1 min, extension at 72°C for 1 min, and a final extension of 10 min at 72°C. PCR products along with a 100 bp DNA ladder (Invitrogen, USA) were separated on 1.5% agarose gels in 1X TAE buffer stained with ethidium bromide (0.5 µg/mL) and visualized under ultraviolet light in an Axygen™ Gel documentation system (Corning, USA).

### 2.5. Antimicrobial Resistance Profiling

AMR profiling of the isolates was performed by using eight antibiotics of seven classes on MHA agar as described earlier [19]. In brief, overnight-grown bacterial inoculums were adjusted to 0.5 McFarland standards and swabbed on preincubated MHA plates by sterile cotton swabs and left for 10–15 minutes to dry. Then, standard antibiotic discs were placed on MHA plates with sterile forceps and incubated aerobically at 37°C for 24 h. Following incubation, the organisms were categorized as “resistant” or “susceptible” based on their diameter of zone of inhibition according to CLSI guidelines [20]. Antibiotic classes and antibiotics used in this study included aminoglycosides (gentamicin- 10 µg and streptomycin- 10 µg), fluoroquinolones (ciprofloxacin- 5 µg), *β*-lactam (ampicillin- 10 µg), tetracyclines (tetracycline- 30 µg), phenicols (chloramphenicol- 30 µg), sulfonamides (trimethoprim-sulfamethoxazole- 1.25/23.75 *μ*g and sulfonamide-300 µg), and macrolides (erythromycin- 15 µg). *E. coli* ATCC 25922 was used as a quality control strain for the interpretation of the antimicrobial susceptibility test results.

### 2.6. Detection of Antimicrobial Resistance Genes (ARGs) in the Isolates

The presence of following antibiotic resistance genes associated to streptomycin (*aad*A1), tetracycline (*tet*(A) and *tet*(B)), trimethoprim (*dfr*A1), quinolones (*qnr*A), gentamicin (*aac*(3)-IV), streptomycin (*aadA1)*, sulfonamide (*sul*1), betalactams (*bla*_SHV_ and CITM), erythromycin (*ere*(A)), and chloramphenicol (*cat*A1 and *cml*A) were investigated in all UPEC isolates by PCR using previously described primers [21–24]. Details of the ARGs, their primer sequences, annealing temperature, PCR product size, etc. are presented in Table 1. Basic thermal conditions were initial denaturation for 5 min at 95°C, 35 cycles consisting of denaturation for 1 min at 94°C, annealing for 40 s at the temperature of each respective gene, and extension for 1 min at 72°C, followed by a final extension step of 10 min at 72°C. The annealing temperature varied for each gene (Table 1).

### 2.7. Statistical Analysis

Descriptive and association-based statistical analyses were conducted using Microsoft^®^ Excel v.13.0 and GraphPad Prism v.8.0 statistical tools, respectively. The association between specific AMR phenotypes and the ARG was calculated, and the association was considered significant at a *p*value of <0.05 and was reported as an odds ratio (OR) with 95% confidence intervals (CI). An OR of >1 was considered as positive association or the increasing probability of the cooccurrence of the genotype or phenotype, while an OR of <1 was considered as negative association or the decreasing probability of the cooccurrence of the genotype or phenotype. The degree of agreement between phenotypic and genotypic associations was assessed by Kappa coefficients (*κ*) according to Landis and Koch [25].

## 3. Results

### 3.1. Characteristics of UTI Patients

Out of 83 urine samples investigated in this study, UPEC was isolated from 50.60% (42/83) of samples. The demographic and clinical characteristics of these forty-two UPEC associated UTI patients are presented in Table 2. Among the patients, 19.05% (8/42) were male and 80.95% (34/42) were female within an age range of 13–68 (average ∼35) years. However, the average age of males and females was 42 years and 33 years, respectively. Most of the cases (38.10%) were found in the age group of 21–30 years. About 97.62% and 80.68% of the patients reported uneasy feelings and a burning sense during micturition, respectively. Other commonly reported symptoms include lower abdominal pain (42.86%), fever (83.33%), and back pain (9.52%). A history of recurrent symptoms in the past six months and antibiotic use in the last one month were found in 19.05% and 40.48% of the cases, respectively.

### 3.2. Bacterial Identification and Antimicrobial Resistance Phenotypes

A total of 42 UPEC isolates were used in this study. UPEC isolates from UTI cases were provisionally identified based on cultural, morphological, and biochemical properties. The isolates caused betahemolysis on blood agar, produced greenish black colonies with a metallic sheen on EMB agar and bright, pink colored, raised colonies on MacConkey agar plate, and were single or paired Gram-negative bacilli. In addition, all the isolates fermented dextrose, sucrose, lactose, maltose, and mannitol showed positive results for catalase, indole, and methyl red tests and were negative for the Vogues–Proskauer test. Further confirmation of the isolates as *E. coli* was done by amplifying a 584 bp 16S rRNA gene fragment with specific primers. The antimicrobial resistance profiles of the isolates were investigated and the results are presented in Table 3. Among the isolates, the highest resistance was recorded for tetracycline (92.86%), followed by sulfonamide (71.43%) and ampicillin (52.38%). Only 26.19% and 11.91% of the isolates were found resistant to gentamicin and ciprofloxacin, respectively. None of the isolates were found sensitive to all of the antibiotics used. About 33.33%, 26.19%, and 7.14% of the isolates showed resistance to four, five, and six classes of antibiotics used in this study, respectively (Table 4).

### 3.3. Antimicrobial Resistance Genes (ARGs)

The genes responsible for numerous antibiotic resistance determinants have been investigated, and the results are presented in Table 3. Tetracycline efflux genes such as *tet*(A) (78.57%) and *tet*(B) (76.19%) were the most prevalent resistance genes. Both *tet*(A) and *tet*(B) genes were found in 54.76% (23/42) of the isolates. The second most prevalent resistance gene was *sul*1, which was found in 61.91% of the UPEC. All of the isolates were found to carry one to six of the 12 ARGs, and 28.57% of the UPEC isolates were found to carry five ARGs. On the other hand, six ARGs were detected in 2.38% of the isolates (Table 4).

### 3.4. Associations among Antimicrobial Resistance Phenotypes

Phenotypic resistance of a drug was found to be associated with the phenotypic resistance of another drug. Both positive (OR > 1) and negative (OR < 1) associations were observed in the present study. Resistance to streptomycin was found to be positively associated with resistance to erythromycin (OR: 7; 95% CI: 1.57–31.26, *p*=0.01). Positive associations were also found in the cases of the following pairs of AMR phenotypes: Amp-Gen, Amp-Chl, Amp-TriS, Amp-Sul; Gen-Str; Str-Cip; Cip-TriS, Cip-Ery; Tet-TriS; and Chl-TriS, Chl-Sul, Chl-Ery; but nonsignificant (*p* > 0.05). On the other hand significant negative associations were found in case of Amp-Cip (OR: 0.11, 95% CI: 0.01–1.03, *p*=0.05), Cip-Tet (OR: 0.07, 95% CI: 0.01–0.97, *p*=0.05), and Sul-Ery (OR: 0.25, 95% CI: 0.06–1.06, *p*=0.05) pairs of antibiotics (Supplementary Table 1S).

### 3.5. Associations among Antimicrobial Resistance Genes (ARGs)

Both positive (OR > 1) and negative (OR < 1) associations of ARGs were observed in the present study (Supplementary Table 2S). Presence of *cat*A1 gene was found to be positively associated with the *cml*A gene (OR: 33, 95% CI: 2.92–372.83, *p*=0.01). Positive associations were also observed between gene pairs of CITM-*bla*_SHV_ (OR: 17.14, 95% CI: 1.65–178.09, *p*=0.02), CITM-*dfr*A1 (OR: 9.46, 95% CI: 0.95–94.49, *p*=0.05), and *bla*_SHV_-*dfr*A1 (OR: 18.75, 95% CI: 3.19–110.35, *p*=0.01). On the other hand significant negative associations were observed between CITM-*tet(B)* (OR: 0.05, 95% CI: 0.01–0.51, *p*=0.01) and *bla*_SHV_-*tet*(B) (OR: 0.12, 95% CI: 0.03–0.60, *p*=0.01) gene pairs. In addition, *tet*(A)*, sul*1*, ere*(A)*, aac*(3)-IV*, aad*A1, and *qnrA* genes were not found to be associated with any of the other genes investigated.

### 3.6. Associations between Antimicrobial Resistance Phenotypes and Genotypes

In general, the agreement between resistance associations was found ranging from slight (*κ* = 0.05) for tetracycline to substantial (*κ* = 0.68) for sulfonamide. However, several significant positive associations (*p* < 0.05) were found among AMR phenotypes and the corresponding resistance genes (Table 5). The strongest observed associations were between the following pairs: sulfonamide-*sul*1 (OR:55, CI: 5.73–527.66, *p* ≤ 0.001, *κ* = 0.68), trimethoprime-sulphamethaxole-*dfr*A1 (OR: 49, 95% CI 5.31–452.24, *p* ≤ 0.001, *κ* = 0.66), ciprofloxacin-*qnrA* (OR: 45.33, 95% CI: 3.76–546.25, *p*=0.002, *κ* = 0.61), ampicillin-*bla*_SHV_ (OR: 41.0, 95% CI: 2.20–761.79, *p*=0.012, *κ* = 0.49), chloramphenicol-*cml*A (OR: 14.0, 95% CI: 2.22–87.02, *p*=0.004, *κ* = 0.48), and chloramphenicol-*cat*A1 (OR: 14.5, 95% CI: 1.42–148.57, *p*=0.024, *κ* = 0.36). In some cases, the associations were found nonsignificant (*p* > 0.05) although OR were >1. These include ampicillin-CITM, gentamicin-*aac*(3)-IV, streptomycin*-aad*A1, tetracycline-*tet*(A), tetracycline-*tet*(B), and erythromycin-*ere*(A) pairs. It is evident that every antimicrobial outcome had some isolates with a resistant phenotype, but without the presence of resistance gene (s). Conversely, we have found some isolates that have a resistance gene but are phenotypically not resistant to the corresponding antibiotic. Hence, we could not find a perfect agreement for any of the outcomes.

## 4. Discussion

Resistance to antimicrobials is a global problem and also an arduous task to tackle due to their selection and coselection phenomena. Therefore, this study addresses the association of phenotypic and genotypic resistance traits among 42 UPEC isolates recovered from clinically confirmed UTI patients. UPECs are reported as the most common cause of UTIs which originate from the distal gut microbiota, being responsible for approximately 90% of global UTI cases [26]. The majority (80.95%) of the UTI cases of the current study were from females (Table 2). Previous studies have also reported a higher prevalence of UTIs in females [13, 27]. Anatomical predisposition of females' urogenital organs or other host factors such as catheterization, pregnancy, sexual activity, obstruction of the urinary tract, etc., have been reported as important causes. In the present study, most UTI cases were found in 21–30 years of age (Table 2). Similar findings were also reported previously from Bangladesh [10, 28] and India [29]. Besides, it was also reported that females at the reproductive age of 14–44 years are more vulnerable to UTI [30]. The history of recurrent infection (19.05%) was also recorded in this study.

Most of the UPEC isolates of this study were found to be resistant to common antibiotics including some of those used for UTI treatment such as trimethoprim-sulfamethoxazole and ciprofloxacin at a variable frequency (Table 3). AMR is mainly an acquired property that can be lost or evolved at any time. For this reason, the resistance properties presented by some pathogens to a particular drug may vary temporally and geographically. International surveillance have already warned about the increasing resistance trends of UPEC to trimethoprim-sulfamethoxazole, fluoroquinolones, and other commonly prescribed antibiotics along with the emergence of MDR-UPEC strains [31].

Genes associated with resistance to different antimicrobial agents were also detected in variable frequencies (Table 3). Even multiple genes, as well as resistant phenotypes to multiple antibiotics, were detected in UPEC isolates (Table 4). Our results agree with published reports from Bangladesh and Saudi Arabia [10, 32]. Hossain et al. [10] examined 70 catheter-associated UPEC isolates from UTI patients and reported that about 26% of the isolates were resistant to seven out of ten of the antibiotics tested. In a recent study from Saudi Arabia, it was reported that about 78.0% and 69.0% of the UPEC isolates were found resistant to four and five antibiotics, respectively [32]. A unique challenge for clinical microbiologists, clinicians, and infection control professionals is to deal with extended-spectrum betalactamase (ESBL) producing pathogens. In this study, betalactamase gene, *bla*_SHV_ was found in 26.19% of the UPEC isolates (Table 3). About 11.91% of the isolates were found resistant to ciprofloxacin, a good choice for UTI treatment. Previous studies also reported the emergence of fluoroquinolone resistance in UPEC strains due to easy transfer of the *qnr* genes located on a Tn-like sequence or integron on a conjugative plasmid [33, 34]. Although the prevalence of the UPEC isolates resistant to betalactam and fluoroquinolones was low, the bacterial strains carrying *bla*_SHV_ and *qnr*A genes could not be ignored.

The use of a particular antimicrobial agent can select for its own resistance, as well as serve as a coselection marker for other antimicrobial agents. Even so, sometimes it can also be seen in completely unrelated drug classes [35]. For instances, the use of chloramphenicol for the UTI treatment is very rare in the clinical settings. However, about 28.57% of the isolates showed resistance to chloramphenicol. Moreover, chloramphenicol resistance genes such as *cat*A1 and *cml*A were detected in 11.91% and 19.05% of the isolates, respectively (Tables 3 and 4). Resistance to chloramphenicol might be due to the coselection dynamics among chloramphenicol, oxytetracycline, and sulfamethoxazole as reported earlier [35, 36]. A negative association between *tet*(A) and *tet*(B) resistance genes among UPEC isolates was observed (Supplementary Table 2S). Similar findings were also reported previously, and an incompatibility of plasmids carrying the tetracycline resistance determinants has been attributed to this type of negative association [36]. However, further characterization of the relationships among the resistance gene(s) and the probable link to antimicrobial exposure needs to be investigated. The current effort to limit the emergence or spread of drug-resistance pathogens based on the restrictive use of antimicrobials may not prevent the coselection of genes conferring resistance to unrelated antimicrobial drugs from being used. Therefore, assessments of resistance patterns at the genetic level and the association between ARGs might be critical in AMR surveillance, and for the development of predictive models for the control of drug-resistance phenomena.

Positive associations (OR > 1) between AMR phenotypes and genotypes with and without statistical significance were observed (Table 5). The agreement among the resistance determinants was slight to substantial without any perfect agreement as in the previous studies [37–39]. In *E. coli*, the AMR phenotypic-genotypic agreement of 33% to 85% [39] has been reported for different antimicrobial agents and related genes. However, in our study, it was found that many UPEC isolates with a resistance phenotype lacked the ARGs tested, indicating the occurrence of mutligene mediated AMR. Similarly, some isolates harbored the resistance genes but were phenotypically not resistant to the corresponding antibiotics used in this study. The occurrence of similar AMR phenomena was also reported previously [39]. A possible explanation to such resistance mechanisms is that the phenotypes may be expressed upon the stimulation of many different genetic factors, and that each factor may present a unique epidemiological character [39]. Sometimes, the phenotype or the genotype alone is unable to accurately predict the outcome of the other, as molecular mechanisms of AMR are multifaceted. Thus, the presence or absence of a specific gene corresponding to a particular phenotype does not necessarily imply that the particular strain is resistant or susceptible [40]. The differences between the genotype and phenotype observed in this study might be due to not testing for all possible resistance genes, or genes not being turned on, or the presence of ‘silent gene cassettes' in certain isolates. In addition, phenotypic sensitivity tests might falsely categorize an isolate as ‘susceptible' if the test breakpoint value is much higher than the resistance imparted by the corresponding resistance gene, as in the case of the streptomycin resistance of *E. coli* [41]. Besides, some resistance phenotypes might be associated with point mutations rather than gene acquisition; therefore, no associated resistance gene would be expected [39]. Therefore, in future studies, the search for resistance determinants should not only be limited to phenotypically resistant isolates, but also take susceptible isolates into consideration to trace future emergence of new resistance pheno- and/geno-types. Inclusion of small sample size, few antibiotic resistance genes, nonconfirmation of ESBL producer and SHV gene by sequencing, etc., are the limitations of this study. In-depth investigation would provide broader insights into the association and coselection dynamics of antimicrobial resistance among clinically important pathogens.

## 5. Conclusions

The present study has investigated the AMR and ARG prevalence in UPEC isolates at both phenotypic and genetic levels. Furthermore, the association and coselection phenomena among different antimicrobial classes were studied using a simple statistical approach. Continuous surveillance is necessary to understand the resistance dynamics. A large-scale study using this approach can help to accurately predict the outcome of a specific antibiotic's use on the resistance to other antimicrobial agents as well as their coselection dynamics.

## Figures and Tables

**Table 1:** Primers used to detect antimicrobial resistance genes (ARGs) in uropathogenic *E. coli* isolates.

Antimicrobial class, agent	ARGs	Primer sequence (5′- 3′)	Annealing temperature (^o^C)	PCR product size (bp)	References
**Betalactam** Ampicillin	CITM	F^*∗*^- TGGCCAGAACTGACAGGCAAA	55	462	[21]
		R^*∗∗*^-TTTCTCCTGAACGTGGCTGGC			
	*bla*_SHV_	F-TCGCCTGTGTATTATCTCCC	52	768	
		R-CGCAGATAAATCACCACAATG			
**Aminoglycosides** Gentamicin	*aac*(3)-IV	F-CTTCAGGATGGCAAGTTGGT	55	286	
		R-TCATCTCGTTCTCCGCTCAT			
Streptomycin	*aad*A1	F-TATCCAGCTAAGCGCGAACT	58	447	[22]
		R-ATTTGCCGACTACCTTGGTC			
**Quinolones** Ciprofloxacin	*qnrA*	F-GGGTATGGATATTATTGATAAAG	53	670	[23]
		R-CTAATCCGGCAGCACTATTA			
**Tetracyclines** Tetracycline	*tet*(A)	F-GGTTCACTCGAACGACGTCA	56	577	[22]
		R-CTGTCCGACAAGTTGCATGA			
	*tet*(B)	F–CCTCAGCTTCTCAACGCGTG	56	634	
		R-GCACCTTGCTCATGACTCTT			
**Phenicols** Chloramphenicol	*cat*A1	F-AGTTGCTCAATGTACCTATAACC	55	547	[21]
		R-TTGTAATTCATTAAGCATTCTGCC			
	*cml*A	F-CCGCCACGGTGTTGTTGTTATC	55	698	
		R-CACCTTGCCTGCCCATCATTAG			
**Sulfonamides** Trimethoprim- sulfamethoxazole	*dfr*A1	F-GGAGTGCCAAAGGTGAACAGC	50	367	[24]
		R-GAGGCGAAGTCTTGGGTAAAAAC			
Sulfonamide	*sul*1	F-TTCGGCATTCTGAATCTCAC	50	822	[21]
		R-ATGATCTAACCCTCGGTCTC			
**Macrolides** Erythromycin	*ere*(A)	F-GCCGGTGCTCATGAACTTGAG	52	419	
		R-CGACTCTATTCGATCAGAGGC			

^*∗*^ *F* = forward; ^*∗∗*^*R* = reverse.

**Table 2:** Categorization and clinical symptoms of urinary tract infection patients (*n* = 42).

Characteristics	Prevalence
Mean age irrespective of sex (years/range of year)	35/13–68
Mean age of males (years/range of year)	42/14–68
Mean age of females (years/range of year)	33/13–61
Male (number/% of the patients)	8/19.05
Female (number/% of the patients)	34/80.95
Age distribution of cases (number/% of the patients)	
11–20 years	7/16.67
21–30 years	16/38.10
31–40 years	4/9.52
41–50 years	5/11.91
51–60 years	8/19.05
61–70 years	2/4.75
Clinical conditions general sense of uneasy feeling (number/% of the patients)	41/97.62
Discomfort during micturition (number/% of the patients)	32/76.19
Lower abdominal pain (number/% of the patients)	18/42.86
Burning feelings during micturition (number/% of the patients)	34/80.95
Fever (number/% of the patients)	35/83.33
Back pain (number/% of the patients)	4/9.52
Renal stone (number/% of the patients)	2/4.76
Ulcer (number/% of the patients)	1/2.38
History of recurrent symptoms in the last six months (number/% of the patients)	8/19.05
Antibiotic use in the last one month (number/% of the patients)	17/40.48

**Table 3:** Phenotypic antimicrobial resistance and the presence of antimicrobial resistance genes in uropathogenic *E. coli* isolates (*n* = 42).

Antibiotic class	Antibiotics	Resistant phenotypically (N/%)^*∗*^	ARGs	Resistant genetically (N/%)^†^
Betalactam	Ampicillin	22/52.38	CITM *bla*_SHV_	5/11.91 11/26.19
Aminoglycosides	Gentamicin	11/26.19	*aac*(3)-IV	2/4.76
	Streptomycin	12/28.57	*aad*A1	3/7.14
Fluoroquinolones	Ciprofloxacin	5/11.91	*qnrA*	5/11.91
Tetracyclines	Tetracycline	39/92.86	*tet*(A)	33/78.57
			*tet*(B)	32/76.19
Phenicols	Chloramphenicol	12/28.57	*cat*A1	5/11.91
			*cml*A	8/19.05
Sulfonamides	Trimethoprim-sulfamethoxazole	20/47.62	*dfr*A1	15/35.71
	Sulfonamide	30/71.43	*sul*1	26/61.91
Macrolides	Erythromycin	12/28.57	*ere*(A)	3/7.14

^*∗*^Number/percent of *E. coli* resistant and ^†^Number/percent of the *E. coli* carrying resistance gene.

**Table 4:** Distribution of antimicrobial resistance phenotypes and antimicrobial resistance genes (ARGs) in uropathogenic *E. coli* isolates (*n* = 42).

Number of antibiotic class/ARGs	Phenotypic resistance (N/%)	ARGs (N/%)
1	1/2.38	1/2.38
2	4/9.52	12/28.57
3	9/21.43	7/16.67
4	14/33.33	9/21.43
5	11/26.19	12/28.57
6	3/7.14	1/2.38

**Table 5:** Comparative association of antimicrobial resistance in uropathogenic *E. coli* isolates according to phenotypic and genotypic results.

Antimicrobial agents	Characteristics of isolate					Agreement between phenotypic and genotypic resistance^¶^ (*n* = 42)			
	NP^*∗*^	ARGs	NG^†^	P+/G-^‡^	P-/G+^§^	OR	95% CI	*p*	*κ*
Ampicillin	22	CITM	5	18	1	4.22	0.43–41.45	0.216	0.13
	22	*bla*_SHV_	11	11	0	41	2.20–761.79	0.012	0.49
Gentamicin	11	*aac*(3)-IV	2	10	1	3.00	0.17–52.53	0.451	0.08
Streptomycin	12	*aad*A1	3	10	1	5.8	0.47–71.07	0.169	0.17
Ciprofloxacin	7	*qnrA*	5	3	1	45.33	3.76–546.25	0.002	0.61
Tetracycline	39	*tet*(A)	33	8	2	1.94	0.16–24.16	0.607	0.07
		*tet*(B)	32	9	2	1.67	0.13–20.58	0.690	0.05
Chloramphenicol	12	*cat*A1	5	8	1	14.5	1.42–148.57	0.024	0.36
		*cml*A	8	6	2	14	2.25–87.02	0.004	0.48
Trimethoprim-sulphamethaxole	20	*dfr*A1	15	6	1	49	5.31–452.24	<0.001	0.66
Sulfonamide	30	*sul*1	26	5	1	55	5.73–527.66	<0.001	0.68
Erythromycin	12	*ere*(A)	3	10	1	5.8	0.47–71.07	0.169	0.17

^*∗*^ *NP*, number of *E. coli* expressing phenotypic resistance to the indicated antimicrobial agent. ^†^*NG*, number of *E. coli* carrying the indicated antimicrobial resistance gene. ^‡^*P*+/G-, number of phenotypically resistant *E. coli* (*P*+) with no resistance gene (G-) for the drug tested. ^*§*^P-/G+, number of phenotypically susceptible *E. coli* (P-) with a resistance gene (G+) for the drug tested. ^¶^Association between AMR phenotypes and ARGs and phenotype-genotype intertest agreement. *n*, number of *E. coli*.

## Data Availability

The data used to support the findings of this study are included within the article.

## Abbreviations

AMR: Antimicrobial resistance
ARGs: Antimicrobial resistance genes
CI: Confidence interval
CLSI: Clinical laboratory standards institute
MDR: Multidrug resistance
MHA: Mueller–Hinton agar
OR: Odds ratio
UPEC: Uropathogenic *Escherichia coli.*

## Supplementary Materials

Supplementary Materials Table 1S: pairwise statistical association between antimicrobial resistance (AMR) phenotypes of uropathogenic *E. coli* isolates. Table 2S: pairwise statistical associations between antimicrobial resistance genes (ARGs) of uropathogenic *E. coli* isolates.
