# Supplementary material for: Association of Antibiotic Resistance Traits in Uropathogenic Escherichia coli (UPEC) Isolates
Source: Can J Infect Dis Med Microbiol. 2022 Mar 16;2022:4251486. doi: 10.1155/2022/4251486 (PMC8942690; doi:10.1155/2022/4251486)
Supplement: Supplementary Materials — Table 1S: pairwise statistical association between antimicrobial resistance (AMR) phenotypes of uropathogenic E. coli isolates. Table 2S: pairwise statistical associations between antimicrobial resistance genes (ARGs) of uropathogenic E. coli isolates. [file 4251486.f1.docx]

**Supplementary Table**

**Table 1S: Pairwise statistical association between antimicrobial resistance (AMR) phenotypes of uropathogenic *E. coli* isolates^*^**

| **Predictor**  **Outcome** | **Amp** | **Gen** | **Str** | **Cip** | **Tet** | **Chl** | **TriS** | **Sul** | **Ery** |
| --- | --- | --- | --- | --- | --- | --- | --- | --- | --- |
| Amp** |  | 1.13  (0.28- 4.47)  0.87 | 0.55 (0.14-2.10)  0.38 | 0.11 (0.01-1.03)  0.05 | 0.53 (0.04-6.29)  0.61 | 1.4  (0.36- 5.41)  0.63 | 2.23  (0.64- 7.74)  0.21 | 1.83 (0.47- 7.10) 0.38 | 0.33 (0.08-1.36) 0.13 |
| Gen |  |  | 1.64 (0.38- 7.13) 0.51 | 0.42  (0.04- 3.92) 0.44 | 0.69 (0.06- 8.45) 0.77 | 0.92 (0.20- 4.26) 0.91 | 0.54 (0.13- 2.20) 0.39 | 0.61 (0.14- 2.64) 0.51 | 0.92 (0.20- 4.26) 0.91 |
| Str |  |  |  | 2.17 (0.41-11.60) 0.37 | 0.79 (0.07- 9.57) 0.75 | 0.78 (0.17- 3.56) 0.75 | 0.71 (0.19- 2.76) 0.63 | 0.43 (0.10-1.77) 0.24 | 7.00 (1.57-31.26) 0.01 |
| Cip |  |  |  |  | 0.07 (0.01- 0.97) 0.05 | 0.36 (0.04- 3.40) 0.38 | 1.58 (0.31- 8.15) 0.58 | 0.22 (0.04-1.21) 0.08 | 4.50 (0.83-24.44) 0.08 |
| Tet |  |  |  |  |  | 0.79 (0.07-9.57) 0.85 | 7.36 (0.36-151.92) 0.20 | 0.31 (0.02-6.56) 0.46 | 0.79 (0.07-9.57) 0.85 |
| Chl |  |  |  |  |  |  | 1.14 (0.30-4.36) 0.85 | 2.50 (0.46-13.65) 0.29 | 1.38 (0.32-5.85) 0.67 |
| TriS |  |  |  |  |  |  |  | ND | 0.44 (0.11-1.77) 0.25 |
| Sul |  |  |  |  |  |  | ND |  | 0.25 (0.06-1.06) 0.05 |

^*^Values in each cell: In front of parenthesis is odds ratio (OR); within the parenthesis is 95% CI and after parenthesis is *p*-value. ND: Not done

**Amp: Ampicillin; Gen: Gentamicin; Str: Streptomycin; Cip: Ciprofloxacin; Tet: Tetracycline; Chl: Chloramphenicol; TriS: Trimethoprim-Sulfamethoxazole; Sul: Sulfonamide.

**Supplementary Table**

**Table 2S: Pairwise statistical associations between antimicrobial resistance genes (ARGs) of uropathogenic *E.* coli isolates^*^**

| **Predictor**  **Outcome** | **CITM** | ***bla*_SHV_** | ***aac*(3)-IV** | ***aad*A1** | ***qnrA*** | ***tet*(A)** | ***tet*(B)** | ***cat*A1** | ***cml*A** | ***dfr*A1** | ***sul*1** | ***ere*(A)** |
| --- | --- | --- | --- | --- | --- | --- | --- | --- | --- | --- | --- | --- |
| CITM |  | 17.14 (1.65-178.09)  0.02 | 1.29 (0.05-30.63)  0.87 | 0.90 (0.04-19.83)  0.95 | 0.54 (0.03-11.15)  0.69 | 3.67 (0.19-72.68)  0.39 | 0.05 (0.01-0.51)  0.01 | 2.06 (0.18-23.30)  0.56 | 0.32 (0.02-6.30)  0.45 | 9.46 (0.95-94.49)  0.05 | 2.73 (0.28-26.87)  0.39 | 4.38 (0.32-59.73)  0.27 |
| *bla*_SHV_ |  |  | 0.51 (0.02-11.53)  0.67 | 1.45 (0.12-17.77)  0.77 | 0.21 (0.01-4.11)  0.30 | 3.48 (0.38-31.63)  0.27 | 0.12 (0.03-0.60)  0.01 | 0.21 (0.01-4.11)  0.30 | 0.93 (0.16-5.45)  0.93 | 18.75 (3.19-110.35)  0.001 | 3.71 (0.69-20.04)  0.13 | 0.35 (0.02-7.41)  0.50 |
| *aac*(3)-IV |  |  |  | 2.14 (0.09-54.06)  0.64 | 1.29 (0.05-30.63)  0.87 | 1.51 (0.07-34.21)  0.80 | 1.72 (0.08-38.83)  0.73 | 1.29 (0.05-30.63)  0.87 | 0.77 (0.03-17.47)  0.87 | 0.32 (0.02-7.31)  0.48 | 0.60 (0.04-10.32)  0.73 | 2.14 (0.09-54.06)  0.64 |
| *aad*A1 |  |  |  |  | 0.90 (0.04-19.82)  0.94 | 2.18 (0.10-46.10)  0.61 | 2.49 (0.12-52.39)  0.56 | 0.90 (0.04-19.83)  0.95 | 0.53 (0.03-11.28)  0.68 | 0.89 (0.07-10.75)  0.92 | 0.28 (0.02-3.37)  0.32 | 1.49 (0.06-35.11)  0.81 |
| *qnrA* |  |  |  |  |  | 0.35 (0.05-2.51)  0.30 | 1.29 (0.13-13.04)  0.83 | 0.54 (0.03-11.15)  0.69 | 0.32 (0.02-6.30)  0.45 | 0.132 (0.01-2.56)  0.18 | 0.91 (0.14-6.16)  0.93 | 4.38 (0.32-59.73)  0.27 |
| *tet*(A) |  |  |  |  |  |  | 0.12 (0.01-2.22)  0.15 | 1.10 (0.11-11.31)  0.93 | 0.77 (0.13-4.72)  0.79 | 5.90 (0.66- 52.70)  0.11 | 1.40 (0.31-6.24)  0.66 | 0.52 (0.04-6.44)  0.61 |
| *tet*(B) |  |  |  |  |  |  |  | 1.61 (0.17-15.63)  0.68 | 0.43 (0.08-2.26)  0.32 | 0.26 (0.06-1.15)  0.08 | 0.13 (0.01-1.11)  0.06 | 2.49 (0.12-52.39)  0.56 |
| *cat*A1 |  |  |  |  |  |  |  |  | 33.0 (2.92-372.83)  0.01 | 1.23 (0.18-8.33)  0.83 | 2.73 (0.28-26.87)  0.39 | 4.38 (0.32-59.73)  0.27 |
| *cml*A |  |  |  |  |  |  |  |  |  | 2.09 (0.44-9.96)  0.35 | 2.10 (0.37-11.96)  0.40 | 0.53 (0.03-11.28)  0.68 |
| *dfr*A1 |  |  |  |  |  |  |  |  |  |  | 2.20 (0.56-8.69)  0.26 | 0.23 (0.01-4.68)  0.34 |
| *sul*1 |  |  |  |  |  |  |  |  |  |  |  | 1.25 (0.10-15.01)  0.86 |

^*^Values in each cell: In front of the parenthesis is odds ratio; within the parenthesis is 95% CI and after parenthesis is *p* value.
